# Metformin Protects against NMDA-Induced Retinal Injury through the MEK/ERK Signaling Pathway in Rats

**DOI:** 10.3390/ijms22094439

**Published:** 2021-04-23

**Authors:** Koki Watanabe, Daiki Asano, Hiroko Ushikubo, Akane Morita, Asami Mori, Kenji Sakamoto, Kunio Ishii, Tsutomu Nakahara

**Affiliations:** 1 Department of Molecular Pharmacology, Kitasato University School of Pharmaceutical Sciences, Tokyo 108-8641, Japan; pp10256.molpharm@gmail.com (K.W.); asanod@pharm.kitasato-u.ac.jp (D.A.); hiroko.sakai@hamayaku.ac.jp (H.U.); moritaa@pharm.kitasato-u.ac.jp (A.M.); mori@pharm.teikyo-u.ac.jp (A.M.); sakamoto@pharm.teikyo-u.ac.jp (K.S.); ishiik@pharm.kitasato-u.ac.jp (K.I.); 2 Center for Pharmaceutical Education, Faculty of Pharmacy, Yokohama University of Pharmacy, Kanagawa 245-0066, Japan; 3 Laboratory of Medical Pharmacology, Department of Clinical & Pharmaceutical Sciences, Faculty of Pharma-Sciences, Teikyo University, Tokyo 173-8605, Japan

**Keywords:** AMP-activated protein kinase, extracellular signal-regulated kinase, glutamate, neurotoxicity, retina

## Abstract

Metformin, an anti-hyperglycemic drug of the biguanide class, exerts positive effects in several non-diabetes-related diseases. In this study, we aimed to examine the protective effects of metformin against *N*-methyl-D-aspartic acid (NMDA)-induced excitotoxic retinal damage in rats and determine the mechanisms of its protective effects. Male Sprague–Dawley rats (7 to 9 weeks old) were used in this study. Following intravitreal injection of NMDA (200 nmol/eye), the number of neuronal cells in the ganglion cell layer and parvalbumin-positive amacrine cells decreased, whereas the number of CD45-positive leukocytes and Iba1-positive microglia increased. Metformin attenuated these NMDA-induced responses. The neuroprotective effect of metformin was abolished by compound C, an inhibitor of AMP-activated protein kinase (AMPK). The AMPK activator, AICAR, exerted a neuroprotective effect in NMDA-induced retinal injury. The MEK1/2 inhibitor, U0126, reduced the neuroprotective effect of metformin. These results suggest that metformin protects against NMDA-induced retinal neurotoxicity through activation of the AMPK and MEK/extracellular signal-regulated kinase (ERK) signaling pathways. This neuroprotective effect could be partially attributable to the inhibitory effects on inflammatory responses.

## 1. Introduction

Metformin, an anti-hyperglycemic drug of the biguanide class, controls blood glucose levels by increasing insulin sensitivity and enhancing glucose uptake in the liver, and is widely used in the treatment of type 2 diabetes mellitus [1]. In addition to its glucose-lowering effect, metformin exerts positive effects in several non-diabetes-related diseases [2]. There is abundant evidence suggesting that these effects of metformin are associated with the activation of the AMP-activated protein kinase (AMPK) system; however, there are data suggesting that metformin acts through AMPK-independent mechanisms [3].

Previous studies have demonstrated several beneficial effects of metformin in various central nervous system disorders [4,5,6]. In the visual system, metformin exerts inhibitory effects on inflammatory responses [7] and angiogenesis [7,8], as well as vasodilatory effects on the retinal blood vessels [9]. Furthermore, metformin protects against retinal neuronal damage in diabetic animals [10,11] and maintains the integrity of the photoreceptors and retinal pigment epithelium, through the activation of AMPK, in animal models of retinitis pigmentosa and age-related macular degeneration [12,13]. Similarly, AICAR (5-aminoimidazole-4-carboxamide-1-β-D-ribofuranoside), an activator of AMPK, attenuates diabetes-induced retinal inflammation [7,14] and dilates retinal blood vessels [9]. Thus, activation of the AMPK system contributes to beneficial effects in the visual system.

Neuronal cell loss associated with glutamate-induced neurotoxicity is involved in the pathogenesis of several ocular diseases including glaucoma [15] and diabetic retinopathy [16]. However, the effect of metformin on glutamate-induced neurotoxicity in the retina remains to be investigated. In many cases, the excessive stimulation of the *N*-methyl-D-aspartic acid (NMDA) receptors contributes to the neurotoxic effect of glutamate [17,18]. Neuroinflammation also contributes to the progression of retinal neuronal damage [19,20,21]. Therefore, in the present study, we examined the effects of metformin against NMDA-induced retinal neurotoxicity in rats and determined how metformin prevents inflammatory responses, such as leukocyte infiltration and microglial activation, in the retina. Activation of the MEK/extracellular signal-regulated kinase (ERK) signaling pathway might be involved in neuroprotection in the retina [22,23,24,25,26]. We also examined the role of the MEK/ERK pathway in the neuroprotective effect of metformin.

## 2. Results

**Figure 1:**
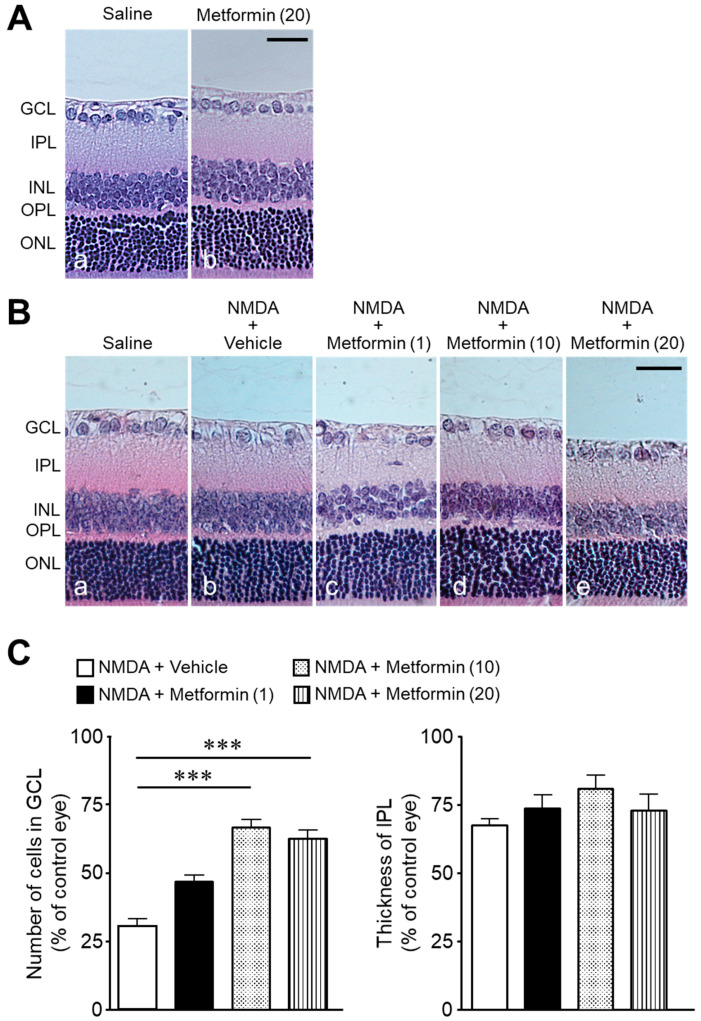
Metformin attenuates NMDA-induced cell loss in the ganglion cell layer (GCL). The number of cells in the GCL and thickness of the inner plexiform layer (IPL) were measured seven days after intravitreal injection. (**A**): Saline (**a**); metformin (20 nmol) (**b**). Scale bar, 30 µm. (**B**): Saline (**a**); NMDA (200 nmol) + vehicle (**b**); NMDA (200 nmol) + metformin (1 nmol) (**c**); NMDA (200 nmol) + metformin (10 nmol) (**d**); NMDA (200 nmol) + metformin (20 nmol) (**e**). Scale bar, 30 µm. (**C**): Bar graph showing the number of cells in the GCL and thickness of the IPL. *n* = 8–15. *** *p* < 0.001; Kruskal–Wallis test.

Intravitreal injection of metformin (20 nmol) had no significant change in cell number in the ganglion cell layer (GCL) compared to controls (92 ± 1 cells/mm in the saline group, *n* = 4, vs., 91 ± 3 cells/mm in the metformin group, *n* = 4, *p* = 0.8088; unpaired t-test) (Figure 1A). In contrast, following intravitreal injection of NMDA (200 nmol/eye), the cell number in the GCL and the thickness of the inner plexiform layer (IPL) decreased compared to that injected with saline alone (Figure 1B(a,b)). The significant increase in the number of cells in the GCL was observed following simultaneous injection of metformin (10 or 20 nmol) with NMDA (Figure 1B(d,e),C). A tendency of reduced IPL thinning was observed but failed to attain statistical significance (Figure 1B,C).

**Figure 2:**
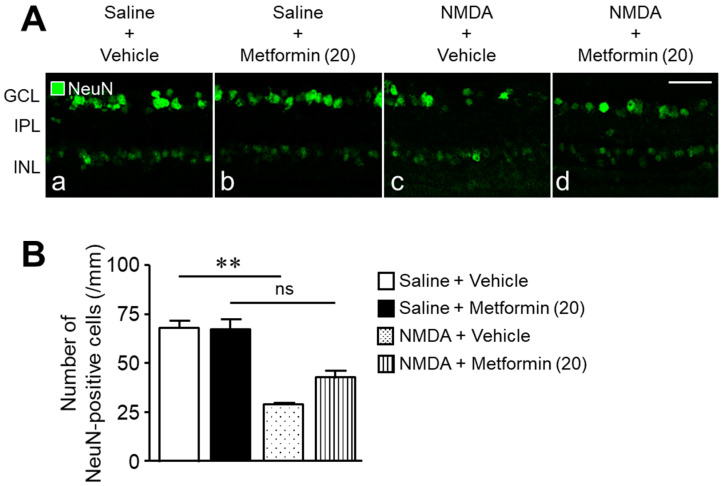
Metformin protects against NMDA-induced loss of NeuN-positive cells. The number of NeuN-positive cells in the ganglion cell layer (GCL) was counted one day after intravitreal injection of NMDA. (**A**): Saline + vehicle (**a**); saline + metformin (**b**); NMDA + vehicle (**c**); NMDA + metformin (**d**). The doses of NMDA and metformin were 200 nmol/eye and 20 nmol/eye, respectively. Scale bar, 50 µm. (**B**): Bar graph showing the number of NeuN-positive cells per millimeter in the GCL of each retina. *n* = 5–6. ** *p* < 0.01; ns: no significance; Kruskal–Wallis test.

Next, we investigated the protective effect of metformin against retinal damage using cross-sections stained with neuron-specific markers, anti-NeuN, and anti-parvalbumin (PV) antibodies. Many NeuN-positive cells were observed in the GCL, and some were also found in the inner nuclear layer (INL) (Figure 2A(a)). Metformin did not significantly decrease the number of NeuN-positive cells compared to the saline + vehicle group (Figure 2A(a,b),B). The number of NeuN-positive cells decreased one day after NMDA injection (Figure 2A(c),B). The simultaneous injection of metformin and NMDA partially rescued the reduction in the NeuN-positive cell number compared to the NMDA + vehicle group (the number of NeuN-positive cells; 30 ± 1 cells in the NMDA + vehicle group, *n* = 5, vs. 43 ± 3 cells in the NMDA + metformin group, *n* = 6, *p* = 0.0031; unpaired t-test, Figure 2A(c,d),B).

**Figure 3:**
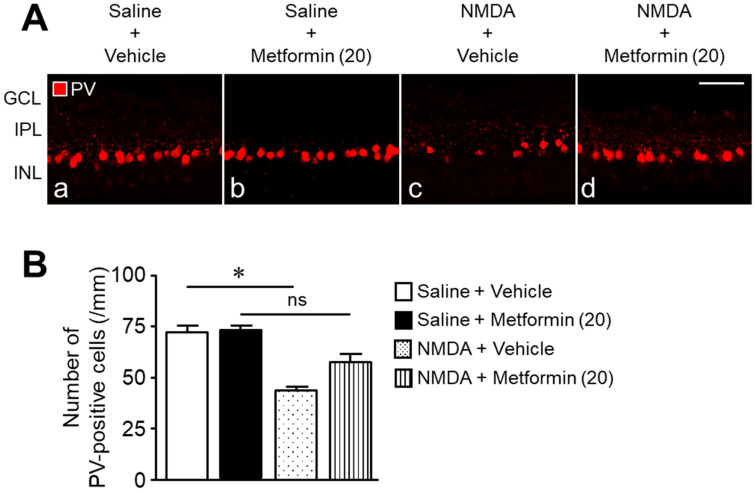
Metformin protects against NMDA-induced loss of parvalbumin (PV)-positive cells. The number of PV-positive cells in the inner nuclear layer (INL) was counted one day after intravitreal injection of NMDA. (**A**): Saline + vehicle (**a**); saline + metformin (**b**); NMDA + vehicle (**c**); NMDA + metformin (**d**). The doses of NMDA and metformin were 200 nmol/eye and 20 nmol/eye, respectively. Scale bar, 50 µm. (**B**): Bar graph showing the number of PV-positive cells per millimeter in the INL of each retina. *n* = 5–6. * *p* < 0.05; ns: no significance; Kruskal–Wallis test.

Next, we examined the effect of metformin (20 nmol) on NMDA-induced loss of PV-positive cells. In control retinas of the saline + vehicle group, PV-positive cells were observed in the inner part of the INL and their processes were found in the IPL (Figure 3A(a)). Parvalbumin is mainly expressed in AII amacrine cells of the INL [27]; therefore, the majority of PV-positive cells most likely represented AII amacrine cells. No significant difference was found in the number of PV-positive amacrine cells between the control group (saline + vehicle) and the group receiving metformin (saline + metformin, Figure 3A(a,b),B). The number of PV-positive amacrine cells decreased one day after NMDA injection (Figure 3A(c),B); however, this phenomenon was partially prevented by the simultaneous injection of metformin and NMDA (Figure 3A(d),B). The number of PV-positive amacrine cells was significantly (*p* = 0.0262; unpaired t-test) higher in the NMDA + metformin group (57 ± 4 cells, *n* = 6) than in the NMDA + vehicle group (44 ± 1 cells, *n* = 5). These results suggest that metformin exerts a protective effect on amacrine cells and on RGCs.

**Figure 4:**
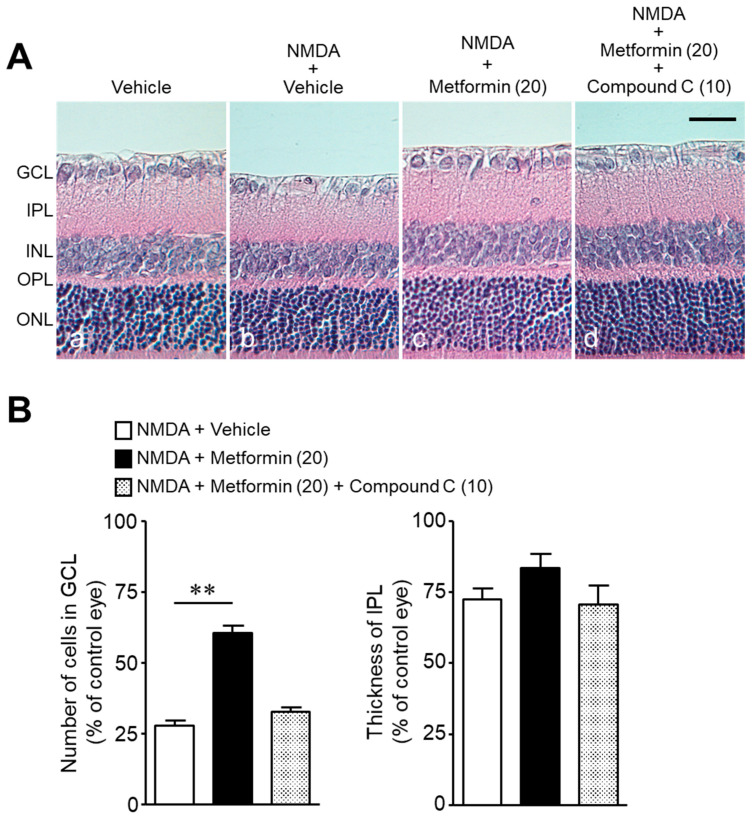
The AMPK inhibitor, compound C, attenuates the protective effects of metformin against NMDA-induced cell loss in the ganglion cell layer (GCL). The number of cells in the GCL and thickness of the inner plexiform layer (IPL) were measured seven days after intravitreal injection. (**A**): Vehicle (DMSO, (**a**); NMDA + vehicle (**b**); NMDA + metformin (**c**); NMDA + metformin + compound C (**d**). The doses of NMDA, metformin, and compound C were 200 nmol/eye, 20 nmol/eye, and 10 nmol/eye, respectively. Scale bar, 30 µm. (**B**): Bar graph showing the number of cells in the GCL and thickness of the IPL. *n* = 6. ** *p* < 0.01; Kruskal–Wallis test.

**Figure 5:**
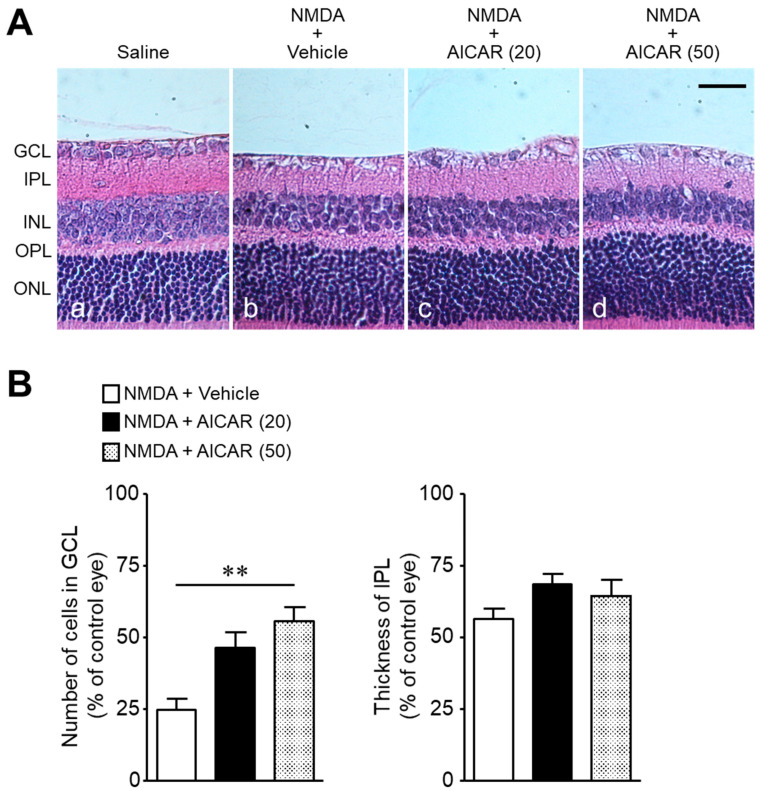
The AMPK activator, AICAR, attenuates NMDA-induced cell loss in the ganglion cell layer (GCL). The number of cells in the GCL and thickness of the inner plexiform layer (IPL) were measured seven days after intravitreal injection. (**A**): Saline (**a**); NMDA (200 nmol) + vehicle (**b**); NMDA (200 nmol) + AICAR (20 nmol) (**c**); NMDA (200 nmol) + AICAR (50 nmol) (**d**). Scale bar, 30 µm. (**B**): Bar graph showing the number of cells in the GCL and thickness of the IPL. *n* = 5–6. ** *p* < 0.01; Kruskal–Wallis test.

We examined whether (1) the protective effect of metformin against NMDA-induced retinal injury is mediated through activation of the AMPK system, and (2) activation of AMPK protects against NMDA-induced retinal injury. The co-administration of the AMPK inhibitor, compound C, with metformin abolished the neuroprotective effect of metformin (Figure 4A,B). As observed after metformin treatment, AICAR, an activator of AMPK, attenuated NMDA-induced cell loss in the GCL without preventing the reduction in IPL thickness (Figure 5A,B). These results suggest that activation of the AMPK system protects against NMDA-induced neurotoxicity.

**Figure 6:**
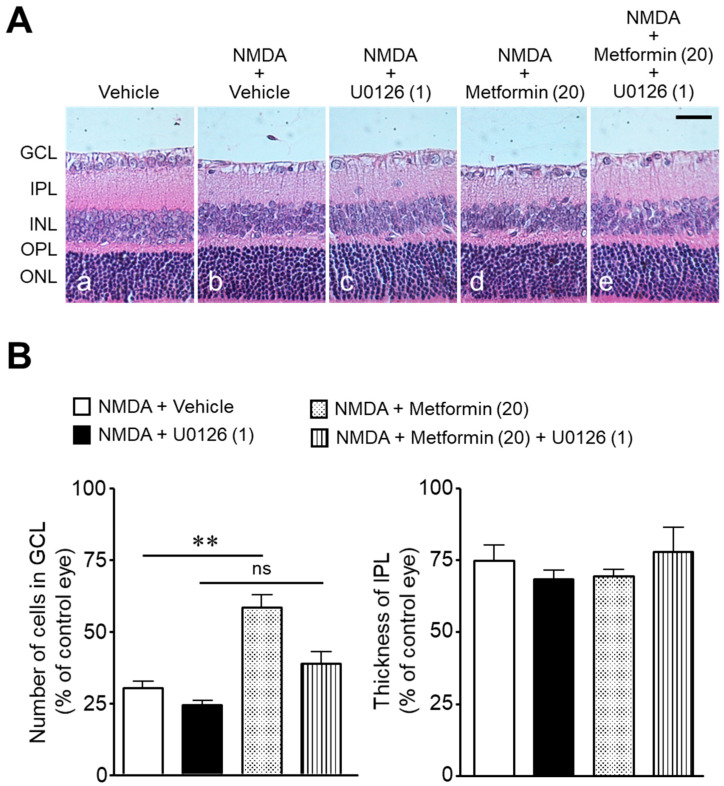
The role of the MEK/ERK signaling pathway in the protective effects of metformin against NMDA-induced cell loss in the ganglion cell layer (GCL). The number of cells in the GCL and thickness of the inner plexiform layer (IPL) were measured seven days after intravitreal injection. (**A**): Vehicle (DMSO, (**a**); NMDA + vehicle (**b**); NMDA + U0126 (**c**); NMDA + metformin (**d**); NMDA + metformin + U0126 (**e**). The doses of NMDA, metformin, and U0126 were 200, 20, and 1 nmol/eye, respectively. Scale bar, 30 µm. (**B**): Bar graph showing the number of cells in the GCL and thickness of the IPL. *n* = 5–10. ** *p* < 0.01; ns: no significance; Kruskal–Wallis test.

To determine whether the neuroprotective effect of metformin was mediated through the MEK/ERK signaling pathway, we examined the effect of U0126, an inhibitor of MEK1/2, on NMDA-induced cell loss in the GCL. The pharmacological inhibition of the MEK/ERK signaling pathway attenuated the neuroprotective effect of metformin (Figure 6).

**Figure 7:**
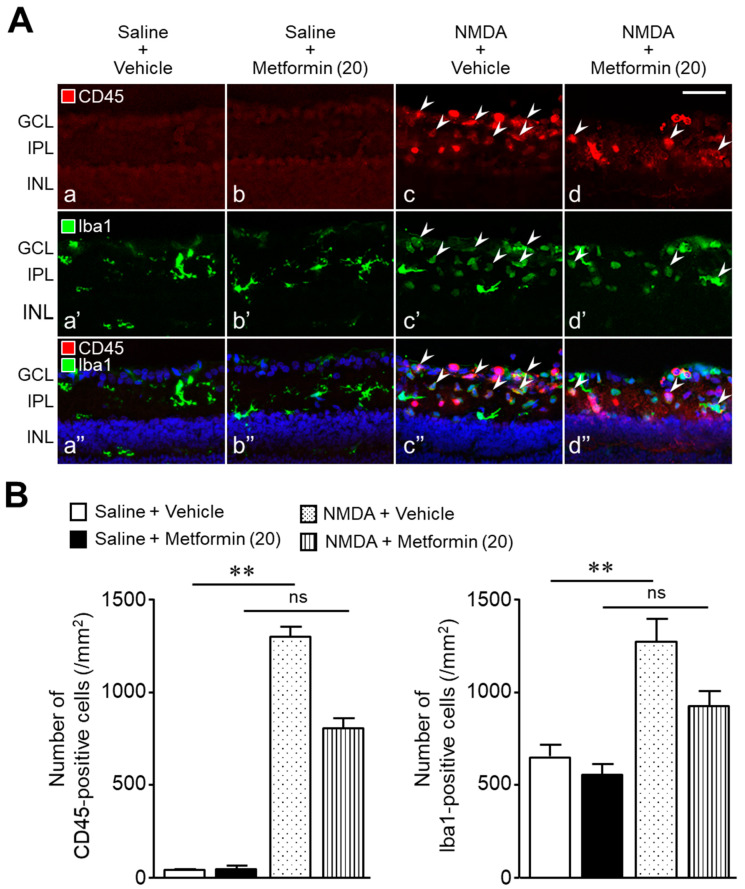
Metformin prevents leukocyte infiltration and microglia activation in the retinas of NMDA-injected eyes. The numbers of CD45-positive cells and Iba1-positive cells were counted one day after intravitreal injection of NMDA. (**A**): Saline + vehicle (**a**,**a’**,**a”**); saline + metformin (**b**,**b’**,**b”**); NMDA + vehicle (**c**,**c’**,**c”**); NMDA + metformin (**d**,**d’**,**d”**). The doses of NMDA and metformin were 200 and 20 nmol/eye, respectively. Arrowheads indicate CD45- and Iba1-double-positive cells. Blue nuclei: 4′,6-diamidino-2-phenylindole (DAPI). Scale bar, 50 µm. (**B**): Bar graphs showing the number of CD45-positive cells and Iba1-positive cells present in the ganglion cell layer (GCL) and inner plexiform layer (IPL) of each retina. *n* = 5–6. ** *p* < 0.01; ns: no significance; Kruskal–Wallis test.

Figure 7 shows the distribution and number of cells positive for CD45 and ionized calcium-binding adaptor molecule 1 (Iba1) in the retina one day after intravitreal NMDA injection. In the saline + vehicle and saline + metformin groups, few CD45-positive cells were observed (Figure 7A(a,b)) and the Iba1-positive microglia exhibited a ramified morphology (Figure 7A(a’,b’)). However, in the NMDA + vehicle group, many CD45-positive cells were observed in the GCL and IPL (Figure 7A(c)). The number of CD45-positive cells was significantly higher in the NMDA + vehicle group than that in the saline + vehicle group (Figure 7B). In the NMDA + vehicle group, Iba1-positive cells exhibited a transformation from the ramified form to an ameboid shape, and the number of Iba1-positive cells also increased compared to the control retinas (saline + vehicle, Figure 7B). Some of the CD45-positive cells were co-stained with anti-Iba1 antibodies in the retinas of NMDA-injected eyes (Figure 7A(c”)). The numbers of CD45-positive cells and Iba1-positive cells in the NMDA + metformin group were significantly lower than those in the NMDA + vehicle group (the number of CD45-positive cells; 1298 ± 55 cells in the NMDA + vehicle group, *n* = 5, vs. 799 ± 60 cells in the NMDA + metformin group, *n* = 6, *p* = 0.0002; unpaired t-test; the number of Iba1-positive cells; 1279 ± 122 cells in the NMDA + vehicle group, *n* = 5, vs. 917 ± 85 cells in the NMDA + metformin group, *n* = 6, *p* = 0.0034; unpaired t-test)(Figure 7A(c–c”,d–d”),B). These results indicate that metformin prevents inflammatory responses, such as leukocyte infiltration and microglial activation, in NMDA-induced retinal injury.

## 3. Discussion

The present study demonstrates that metformin protects against the death of RGCs and amacrine cells in NMDA-induced excitotoxicity. This neuroprotective effect was abolished by the simultaneous injection of compound C, an AMPK inhibitor, and NMDA. In addition, the AMPK activator, AICAR, exerted a neuroprotective effect against NMDA-induced retinal injury. These results suggest that metformin exerts its protective effect through activation of the AMPK system in the retina.

**Figure 8:**
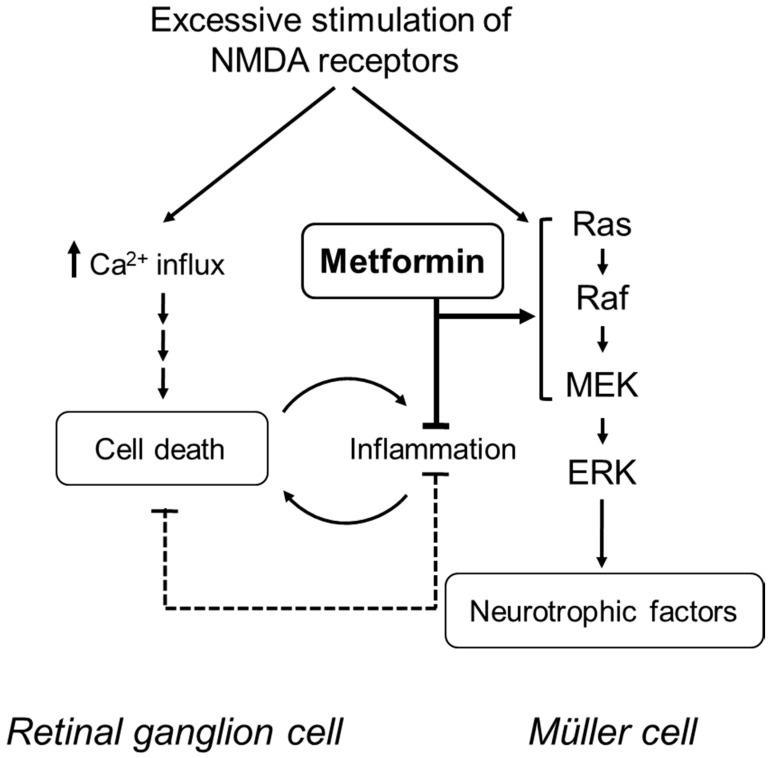
A proposed mechanistic model for the protective effects of metformin in the injured retina. In NMDA-induced neuronal cell death, an increase in the intracellular Ca^2+^ concentration is the key initial step; however, inflammatory responses are also involved. Metformin suppresses the inflammatory responses associated with neuronal cell damage. In addition, metformin may strengthen the endogenous neuroprotective system involving the MEK/ERK signaling pathway.

In NMDA-induced neuronal cell death, the increase in the intracellular Ca^2+^ concentration is the key initial step [28,29]; however, inflammatory responses, such as the upregulation of pro-inflammatory cytokines, infiltration of leukocytes, and activation of microglia in the retina, are also involved [19,20,21]. Nakazawa et al. demonstrated that NMDA-induced retinal injury upregulates intercellular adhesion molecule-1 (ICAM-1), allowing for the adhesion of leukocytes to endothelial cells and the subsequent migration of leukocytes through the interstitial tissue within the retina [19]. Treatment with anti-ICAM-1 antibody suppressed the recruitment of leukocytes and the RGC death following intravitreal injection of NMDA [19]. Metformin has also been shown to suppress the elevated expression of cell adhesion molecules, including ICAM-1, in endothelial cells and to prevent retinal leukocyte adhesion [8,30]. Our present results indicated that metformin suppressed the increase in the number of CD45-positive leukocytes one day after intravitreal injection of NMDA. Similar to the results of leukocyte infiltration, we found that metformin suppressed the increase in the number of Iba-1-positive microglia in the retinas of NMDA-injected eyes. Takeda et al. reported that the pharmacological inhibition and ablation of microglia protects RGCs against NMDA-induced excitotoxicity [20]. Therefore, it is likely that metformin exerts its protective effect through the suppression of inflammatory responses associated with neuronal cell damage in the retinas of NMDA-injected eyes (see Figure 8).

Activation of the MEK/ERK signaling pathway is involved in the survival of retinal neuronal cells in glutamate-induced neurotoxicity [22,23,24,25,26]. In this study, we found that treatment with the MEK1/2 inhibitor, U0126, significantly attenuated metformin-induced protection against NMDA-induced neuronal cell loss. Following intravitreal NMDA injection, activation of ERK occurs earlier than the induction of apoptosis [22,24,25,26]. Therefore, the MEK/ERK signaling pathway may act as an endogenous neuroprotective mechanism. Metformin possibly strengthens the endogenous neuroprotective system involving the MEK/ERK signaling pathway, thereby opposing NMDA-induced retinal neurotoxicity (see Figure 8).

Previous immunohistochemical studies show that the level of phosphorylated ERK (pERK) is enhanced in Müller cells after intravitreal NMDA injection [22,23,24,25,26]. Müller cells are thought to play an important role in the survival of retinal neurons by buffering substances that cause excitotoxicity and by secreting neurotrophic factors [31]. Thus, Müller cells may act as an endogenous neuroprotective system in the retina. Metformin may activate the MEK/ERK pathway and increase the expression of neurotrophic factors in Müller cells. Future studies are needed to clarify this hypothesis.

Our present results suggest that the activation of the MEK/ERK signaling pathway contributes to the neuroprotective effect of metformin against NMDA-induced retinal neuronal cell loss. However, overactivation of NMDA receptors induces the pathologic increase of the Ca^2+^ influx, leading to endoplasmic-reticulum stress [32], oxidative stress [33], and mitochondrial dysfunction [34]. Therefore, further studies are needed to determine how metformin affects these harmful effects in NMDA-induced retinal neurotoxicity.

In summary, our results indicated that metformin protects against NMDA-induced retinal neurotoxicity through the activation of AMPK and that this effect is probably mediated through the activation of the MEK/ERK signaling pathway in the retina. This neuroprotective effect could be partially attributable to the inhibitory effects on inflammatory responses. Glutamate-induced neurotoxicity may be associated with ocular neurodegenerative changes observed in glaucoma [15] and diabetic retinopathy [16]; therefore, metformin could be a potential candidate for neuroprotective interventions in these retinal diseases.

## 4. Materials and Methods

### 4.1. Animals

A total of 133 male Sprague–Dawley rats (7 to 9 weeks old; Charles River Breeding Laboratories, Tokyo, Japan) were used in this study. The rats were housed under controlled conditions of temperature (22 ± 2 °C) and luminosity (12 h light/12 h dark). They were fed with a standard diet (Oriental Yeast Co., Ltd.; Tokyo, Japan), and water was given ad libitum.

### 4.2. Experimental Procedures

#### 4.2.1. Experiment 1

We first examined the effect of metformin on NMDA (200 nmol; Nacalai Tesque, Kyoto, Japan)-induced neuronal cell loss in the retina. To assess the effect of metformin on the damage to retinal neurons, using retinal cross-sections stained with hematoxylin and eosin, 44 rats were divided into four groups as follows: NMDA + vehicle (*n* = 15), NMDA + metformin (1 nmol) (*n* = 8), NMDA + metformin (10 nmol) (*n* = 8), and NMDA + metformin (20 nmol) (*n* = 13). Under general anesthesia with pentobarbital sodium (50 mg/kg; Nacalai Tesque), metformin (1–20 nmol; Tocris Bioscience, Ellisville, MO, USA) or vehicle (saline; vehicle for metformin), mixed with 200 nmol of NMDA (Nacalai Tesque) at a total volume of 5 µL, was injected into the vitreous cavity of one eye of rats. As a control, the same volume of saline was injected into the vitreous cavity of the other eye. In a separate experiment (*n* = 4), the effects of intravitreal injection of metformin (20 nmol) and saline (5 µL) on the retina were compared.

In addition to retinal ganglion cells (RGCs), several amacrine cell types express NMDA receptors in the retina [35,36]; intravitreal injection of NMDA receptors leads to the death of amacrine cells positive for PV [37,38]. Therefore, we next examined the protective effect of metformin on NMDA-induced damage to PV-positive amacrine cells as well as RGCs. In this experiment, 23 rats were divided into four groups as follows: saline (*n* = 6), metformin (20 nmol) (*n* = 6), NMDA + vehicle (*n* = 5), and NMDA + metformin (20 nmol) (*n* = 6). One day after injection, rats were deeply anesthetized with pentobarbital sodium and systemic perfusion was performed with the fixative solution (1% paraformaldehyde (PFA; Nacalai Tesque) in phosphate buffered saline (PBS; Nacalai Tesque) via the aorta. Their eyes were enucleated and processed for immunohistochemistry according to the methods described subsequently (see “4.4. Immunohistochemistry”).

#### 4.2.2. Experiment 2

Next, we examined whether (1) the protective effect of metformin against NMDA-induced retinal injury is mediated through activation of the AMPK system, and (2) activation of AMPK protects against NMDA-induced retinal injury. In the first series of experiments, we examined the effect of compound C, an inhibitor of AMPK, on the effect of metformin on the NMDA (200 nmol)-induced retinal neuronal cell loss in the GCL. In this experiment, 18 rats were divided into three groups as follows: NMDA + vehicle (*n* = 6), NMDA + metformin (20 nmol) (*n* = 6), and NMDA + metformin (20 nmol) + compound C (10 nmol; Abcam Biochemicals, Cambridge, UK) (*n* = 6). NMDA, metformin, and compound C were dissolved in dimethyl sulfoxide (DMSO; Nacalai Tesque). In the second series of experiments, we examined the effect of AICAR (Tocris Bioscience), an activator of AMPK, on NMDA (200 nmol)-induced retinal neuronal cell loss in the GCL. In this experiment, 17 rats were divided into three groups as follows: NMDA + vehicle (*n* = 5), NMDA + AICAR (20 nmol) (*n* = 6), and NMDA + AICAR (50 nmol) (*n* = 6). AICAR was dissolved in saline.

#### 4.2.3. Experiment 3

Activation of the MEK/ERK signaling pathway contributes to the RGC survival in various models of retinal neurodegeneration [22,23,24,25,26]. Therefore, we next examined the effect of U0126, an inhibitor of MEK1/2, on the effect of metformin on NMDA-induced retinal neuronal cell loss in the GCL. In this experiment, 27 rats were divided into the following four groups: NMDA + vehicle (*n* = 7), NMDA + U0126 (1 nmol; Promega, Madison, WI, USA) (*n* = 5), NMDA + metformin (20 nmol) (*n* = 10), and NMDA + metformin (20 nmol) + U0126 (1 nmol) (*n* = 5). NMDA, metformin, and U0126 were dissolved in DMSO. The U0126 dose was selected on the basis of the results of our previous studies [25,26].

#### 4.2.4. Experiment 4

We previously demonstrated that leukocyte infiltration and microglial activation in the retina occurred one day after intravitreal injection of NMDA in rats [26,38,39,40]. Infiltrating leukocytes and activated microglia promote retinal inflammation and exacerbate neuronal damage [19,20]. Therefore, we examined the effects of metformin (20 nmol) on the distribution and the number of CD45 (a leukocyte marker)- and Iba1 (a microglia marker)-positive cells in the retina one day after injection using retinal cross-sections stained with anti-CD45 and anti-Iba1 antibodies. In this experiment, 23 rats were divided into four groups as follows: saline (*n* = 6), metformin (20 nmol) (*n* = 6), NMDA + vehicle (*n* = 5), and NMDA + metformin (20 nmol) (*n* = 6). The immunohistochemical staining of retinal cross-sections was performed as described in the “4.4. Immunohistochemistry” section.

In Experiments 2, 3, and 4, intravitreal injections were administered using the technique described in Experiment 1, wherein the total injection volume used was 5 µL per eye.

### 4.3. Histological Evaluation of the Retina

Histological evaluation was performed according to the methods described in our previous studies [25,26,37,38,40]. Briefly, seven days after injection, rats were deeply anesthetized with pentobarbital sodium. Their eyes were enucleated, fixed with Davidson solution, and embedded in paraffin. Five-micrometer-thick sections were cut through the optic disc of each eye and stained with hematoxylin and eosin. The cell counts in the GCL and the measurement of the IPL thickness were performed in a region 1000 to 1250 μm from the center of the optic nerve head on both sides. The average number of cells for each eye was calculated. The value of the contralateral vehicle (saline or DMSO)-treated control eye was set as 100% versus the drug-treated eye.

### 4.4. Immunohistochemistry

Immunohistochemical staining of retinal cross-sections was performed according to the methods described in our previous studies [26,38,39,40]. Briefly, the eyecups without the cornea and lens were fixed in 1% PFA in PBS overnight at 4 °C and rinsed several times with PBS. The eyecups were incubated in 15% and 30% sucrose in PBS and frozen in optimum cutting temperature (OCT) compound (Sakura Finetek, Torrance, CA, USA). Ocular tissue sections were cut through the optic disc of each eye using a cryostat at a thickness of 16 µm and dried on glass slides. The cross-sections were washed three times in PBS containing 0.3% Triton X100 (PBS-T), incubated in blocking solution (5% normal hamster serum in PBS-T) for 0.5–1 h, and then incubated with primary antibodies diluted in the blocking solution overnight. Primary antibodies used were as follows: rabbit polyclonal anti-PV antibody (1:200, P3088, Sigma-Aldrich, St. Louis, MO, USA), mouse monoclonal anti-CD45 antibody (1:100, 550566, BD Biosciences, San Jose, CA, USA), rabbit polyclonal anti-Iba1 antibody (1:500, 019-19741, Wako, Pure Chemical Industries, Ltd., Osaka, Japan), and rabbit monoclonal anti-NeuN antibody (1:500; D4G40, Cell Signaling Technology, Danvers, MA, USA). Following incubation with primary antibodies, the sections were rinsed with PBS-T and incubated for 4 h with Alexa Fluor 488- or Cy3- conjugated, species-specific secondary antibodies (1:400; Jackson ImmunoResearch Laboratories, West Grove, PA, USA). The sections were then rinsed in PBS-T and mounted using Vectashield mounting medium containing 4′,6-diamidino-2-phenylindole (DAPI) (H-1200, Vector Laboratories, Burlingame, CA, USA).

Two images of the region of the mid-peripheral retina, approximately 1 mm adjacent to the optic nerve on both sides, were obtained from each retinal section. RGC and amacrine cell damage were assessed by counting the NeuN-positive cells in the GCL and PV-positive cells in the INL, respectively. To assess the inflammatory response, the numbers of CD45- and Iba1-positive cells in the GCL and IPL of each retina were counted. The cell number was normalized to the total counting area of the GCL and IPL in each retinal section.

### 4.5. Statistical Analysis

All data are presented as the mean ± standard error (SE). An unpaired t-test and a Kruskal–Wallis test, followed by Dunn′s multiple comparison test, were used to compare values between the two groups and among more than two groups, respectively (Prism 6, GraphPad, San Diego, CA, USA). A P value of less than 0.05 was considered to indicate a statistically significant difference.

## References

[1] Zhou J., Massey S., Story D., Li L. (2018). Metformin: An old drug with new applications. Int. J. Mol. Sci..

[2] Abdelgadir E., Ali R., Rashid F., Bashier A. (2017). Effect of metformin on different non-diabetes related conditions, a special focus on malignant conditions: Review of literature. J. Clin. Med. Res..

[3] Rena G., Hardie D.G., Pearson E.R. (2017). The mechanisms of action of metformin. Diabetologia.

[4] Ashabi G., Khodagholi F., Khalaj L., Goudarzvand M., Nasiri M. (2014). Activation of AMP-activated protein kinase by metformin protects against global cerebral ischemia in male rats: Interference of AMPK/PGC-1α pathway. Metab. Brain Dis..

[5] Porceddu P.F., Ishola I.O., Contu L., Morelli M. (2016). Metformin prevented dopaminergic neurotoxicity induced by 3,4-Methylenedioxymethamphetamine Administration. Neurotox. Res..

[6] Ge X.-H., Zhu G.-J., Geng D.-Q., Zhang H.-Z., He J.-M., Guo A.-Z., Ma L.-L., Yu D.-H. (2017). Metformin protects the brain against ischemia/reperfusion injury through PI3K/Akt1/JNK3 signaling pathways in rats. Physiol. Behav..

[7] Han J., Li Y., Liu X., Zhou T., Sun H., Edwards P., Gao H., Yu F.-S., Qiao X. (2018). Metformin suppresses retinal angiogenesis and inflammation in vitro and in vivo. PLoS ONE.

[8] Joe S.G., Yoon Y.H., Choi J.A., Koh J.-Y. (2015). Anti-angiogenic effect of metformin in mouse oxygen-induced retinopathy is mediated by reducing levels of the vascular endothelial growth factor receptor Flk-1. PLoS ONE.

[9] Mori A., Ishikawa E., Amano T., Sakamoto K., Nakahara T. (2017). Anti-diabetic drug metformin dilates retinal blood vessels through activation of AMP-activated protein kinase in rats. Eur. J. Pharmacol..

[10] Kim Y.S., Kim M., Choi M.Y., Lee D.H., Roh G.S., Kim H.J., Kang S.S., Cho G.J., Kim S.-J., Yoo J.-M. (2017). Metformin protects against retinal cell death in diabetic mice. Biochem. Biophys. Res. Commun..

[11] Nahar N., Mohamed S., Mustapha N.M., Lau S., Ishak N.I.M., Umran N.S. (2021). Metformin attenuated histopathological ocular deteriorations in a streptozotocin-induced hyperglycemic rat model. Naunyn-Schmiedebergs Arch. Pharmacol..

[12] Xu L., Kong L., Wang J., Ash J.D. (2018). Stimulation of AMPK prevents degeneration of photoreceptors and the retinal pigment epithelium. Proc. Natl. Acad. Sci. USA.

[13] Luodan A., Zou T., He J., Chen X., Sun D., Fan X., Xu H. (2019). Rescue of retinal degeneration in rd1 mice by intravitreally injected metformin. Front. Mol. Neurosci..

[14] Kubota S., Ozawa Y., Kurihara T., Sasaki M., Yuki K., Miyake S., Noda K., Ishida S., Tsubota K. (2011). Roles of AMP-activated protein kinase in diabetes-induced retinal inflammation. Investig. Opthalmol. Vis. Sci..

[15] Evangelho K., Mogilevskaya M., Losada-Barragan M., Vargas-Sanchez J.K. (2017). Pathophysiology of primary open-angle glaucoma from a neuroinflammatory and neurotoxicity perspective: A review of the literature. Int. Ophthalmol..

[16] Rossino M.G., Monte M.D., Casini G. (2019). Relationships between neurodegeneration and vascular damage in diabetic retinopathy. Front. Neurosci..

[17] Lam T.T., Siew E., Chu R., Tso M.O. (1997). Ameliorative effect of MK-801 on retinal ischemia. J. Ocul. Pharmacol. Ther..

[18] Solberg Y., Rosner M., Turetz J., Belkin M. (1997). MK-801 has neuroprotective and antiproliferative effects in retinal laser injury. Investig. Ophthalmol. Vis. Sci..

[19] Nakazawa T., Takahashi H., Nishijima K., Shimura M., Fuse N., Tamai M., Hafezi-Moghadam A., Nishida K. (2006). Pitavastatin prevents NMDA-induced retinal ganglion cell death by suppressing leukocyte recruitment. J. Neurochem..

[20] Takeda A., Shinozaki Y., Kashiwagi K., Ohno N., Eto K., Wake H., Nabekura J., Koizumi S. (2018). Microglia mediate non-cell-autonomous cell death of retinal ganglion cells. Glia.

[21] Al-Gayyar M.M.H., Abdelsaid M.A., Matragoon S., Pillai B.A., El-Remessy A.B. (2011). Thioredoxin interacting protein is a novel mediator of retinal inflammation and neurotoxicity. Br. J. Pharmacol..

[22] Hayashi Y., Kitaoka Y., Munemasa Y., Ohtani-Kaneko R., Kikusui T., Uematsu A., Takeda H., Hirata K., Mori Y., Ueno S. (2006). Neuroprotective effect of 17β-estradiol against N-methyl-D-aspartate-induced retinal neurotoxicity via p-ERK induction. J. Neurosci. Res..

[23] Zhou R.-H., Yan H., Wang B.-R., Kuang F., Duan X.-L., Xu Z. (2007). Role of Extracellular Signal-Regulated Kinase in Glutamate-Stimulated Apoptosis of Rat Retinal Ganglion Cells. Curr. Eye Res..

[24] Nakazawa T., Shimura M., Ryu M., Nishida K., Pagès G., Pouysségur J., Endo S. (2007). ERK1 plays a critical protective role againstN-methyl-D-aspartate-induced retinal injury. J. Neurosci. Res..

[25] Ichikawa A., Nakahara T., Kurauchi Y., Mori A., Sakamoto K., Ishii K. (2014). Rapamycin prevents N-methyl-D-aspartate-induced retinal damage through an ERK-dependent mechanism in rats. J. Neurosci. Res..

[26] Hayashi I., Aoki Y., Asano D., Ushikubo H., Mori A., Sakamoto K., Nakahara T., Ishii K. (2015). Protective effects of everolimus against N-methyl-D-aspartic acid-induced retinal damage in rats. Biol. Pharm. Bull..

[27] Wäussle H., Grüunert U., Röhrenbeck J. (1993). Immunocytochemical staining of AII-amacrine cells in the rat retina with antibodies against parvalbumin. J. Comp. Neurol..

[28] Chiu K., Lam T.T., Li W.W.Y., Caprioli J., Kwong J.M.K. (2005). Calpain and N-methyl-d-aspartate (NMDA)-induced excitotoxicity in rat retinas. Brain Res..

[29] Shimazawa M., Suemori S., Inokuchi Y., Matsunaga N., Nakajima Y., Oka T., Yamamoto T., Hara H. (2009). A Novel Calpain Inhibitor, ((1S)-1-((((1S)-1-Benzyl-3-cyclopropylamino-2,3-di-oxopropyl)amino)carbonyl)-3-methylbutyl)carbamic Acid 5-Methoxy-3-oxapentyl Ester (SNJ-1945), Reduces Murine Retinal Cell Death In Vitro and In Vivo. J. Pharmacol. Exp. Ther..

[30] Michiels C.F., Apers S., De Meyer G.R., Martinet W. (2016). Metformin attenuates expression of endothelial cell adhesion molecules and formation of atherosclerotic plaques via autophagy induction. Ann. Clin. Exp. Metab..

[31] Devoldere J., Peynshaert K., De Smedt S.C., Remaut K. (2019). Müller cells as a target for retinal therapy. Drug Discov. Today.

[32] Awai M., Koga T., Inomata Y., Oyadomari S., Gotoh T., Mori M., Tanihara H. (2005). NMDA-induced retinal injury is mediated by an endoplasmic reticulum stress-related protein, CHOP/GADD153. J. Neurochem..

[33] Inokuchi Y., Imai S., Nakajima Y., Shimazawa M., Aihara M., Araie M., Hara H. (2009). Edaravone, a Free Radical Scavenger, Protects against Retinal Damage in Vitro and in Vivo. J. Pharmacol. Exp. Ther..

[34] Marshall J., Wong K.Y., Rupasinghe C.N., Tiwari R., Zhao X., Berberoglu E.D., Sinkler C., Liu J., Lee I., Parang K. (2015). Inhibition of N-Methyl-D-aspartate-induced retinal neuronal death by polyarginine peptides is linked to the attenuation of stress-induced hyperpolarization of the inner mitochondrial membrane potential. J. Biol. Chem..

[35] Gründer T., Kohler K., Kaletta A., Guenther E. (2000). The distribution and developmental regulation of NMDA receptor subunit proteins in the outer and inner retina of the rat. J. Neurobiol..

[36] Zhou Y., Tencerová B., Hartveit E., Veruki M.L. (2016). Functional NMDA receptors are expressed by both AII and A17 amacrine cells in the rod pathway of the mammalian retina. J. Neurophysiol..

[37] Oikawa F., Nakahara T., Akanuma K., Ueda K., Mori A., Sakamoto K., Ishii K. (2012). Protective effects of the β3-adrenoceptor agonist CL316243 against N-methyl-D-aspartate-induced retinal neurotoxicity. Naunyn-Schmiedebergs Arch. Pharmacol..

[38] Naruoka T., Nakahara T., Tsuda Y., Kurauchi Y., Mori A., Sakamoto K., Nishihira J., Ishii K. (2013). ISO-1, a macrophage migration inhibitory factor antagonist, prevents N-methyl-D-aspartate-induced retinal damage. Eur. J. Pharmacol..

[39] Aoki Y., Nakahara T., Asano D., Ushikubo H., Mori A., Sakamoto K., Ishii K. (2015). Preventive effects of rapamycin on inflammation and capillary degeneration in a rat model of NMDA-induced retinal injury. Biol. Pharm. Bull..

[40] Hayashi I., Aoki Y., Ushikubo H., Asano D., Mori A., Sakamoto K., Nakahara T., Ishii K. (2016). Protective effects of PF-4708671 againstN-methyl-d-aspartic acid-induced retinal damage in rats. Fundam. Clin. Pharmacol..

